# Beyond Diet and Exercise: The Impact of Gut Microbiota on Control of Obesity

**DOI:** 10.7759/cureus.49339

**Published:** 2023-11-24

**Authors:** Farah Deeba Kamal, Mehak Dagar, Taufiqa Reza, Alishba Karim Mandokhail, Danyal Bakht, Muhammad Waqas Shahzad, Elizabeth O Silloca-Cabana, Syed Naveed Mohsin, Srikar P Chilla, Syed Faqeer Hussain Bokhari

**Affiliations:** 1 Emergency, Portiuncula University Hospital, Ballinasloe, IRL; 2 Internal Medicine, Himalayan Institute of Medical Sciences, New Delhi, IND; 3 Medicine, Avalon University School of Medicine, Youngstown, USA; 4 Radiology, Quetta Institute of Medical Sciences, Quetta, PAK; 5 Medicine and Surgery, Mayo Hospital, Lahore, PAK; 6 Medicine, Gujranwala Medical College, Gujranwala, PAK; 7 Medicine, Wyckoff Heights Medical Center, Brooklyn, USA; 8 Orthopedics, St. James's Hospital, Dublin, IRL; 9 General Surgery, Cavan General Hospital, Cavan, IRL; 10 Medicine, CARE Hospitals, Hyderabad, IND; 11 Health Sciences, University of East London, London, GBR; 12 Surgery, King Edward Medical University, Lahore, PAK

**Keywords:** appetite regulation, energy metabolism, microbiota-gut-brain axis, gut dysbiosis, inflammation, health issues, energy balance, metabolic disorders, gut microbiota, obesity

## Abstract

Obesity, a widespread health concern characterized by the excessive accumulation of body fat, is a complex condition influenced by genetics, environment, and social determinants. Recent research has increasingly focused on the role of gut microbiota in obesity, highlighting its pivotal involvement in various metabolic processes. The gut microbiota, a diverse community of microorganisms residing in the gastrointestinal tract, interacts with the host in a myriad of ways, impacting energy metabolism, appetite regulation, inflammation, and the gut-brain axis. Dietary choices significantly shape the gut microbiota, with diets high in fat and carbohydrates promoting the growth of harmful bacteria while reducing beneficial microbes. Lifestyle factors, like physical activity and smoking, also influence gut microbiota composition. Antibiotics and medications can disrupt microbial diversity, potentially contributing to obesity. Early-life experiences, including maternal obesity during pregnancy, play a vital role in the developmental origins of obesity. Therapeutic interventions targeting the gut microbiota, including prebiotics, probiotics, fecal microbiota transplantation, bacterial consortium therapy, and precision nutrition, offer promising avenues for reshaping the gut microbiota and positively influencing weight regulation and metabolic health. Clinical applications of microbiota-based therapies are on the horizon, with potential implications for personalized treatments and condition-based interventions. Emerging technologies, such as next-generation sequencing and advanced bioinformatics, empower researchers to identify specific target species for microbiota-based therapeutics, opening new possibilities in healthcare. Despite the promising outlook, microbiota-based therapies face challenges related to microbial selection, safety, and regulatory issues. However, with ongoing research and advances in the field, these challenges can be addressed to unlock the full potential of microbiota-based interventions.

## Introduction and background

Obesity is a chronic metabolic condition that occurs when there is an imbalance between energy intake and expenditure, leading to an excess accumulation of fat, as well as inflammation and metabolic disorders. This condition can result in various health issues, such as respiratory problems, diabetes, cardiovascular diseases, and even cancer. Recent studies have shown that gut microbiota plays a crucial role in the development and progression of obesity and other metabolic disorders [1]. The gut microbiota is a diverse group of microorganisms that reside in the gastrointestinal tract and maintain the overall health of the host. In a state of well-being, the gut microbiota works in harmony with the host, performing essential functions such as nutrient digestion, immune system regulation, and protection against harmful microbes [2]. However, when the gut microbiota becomes imbalanced, known as gut dysbiosis, it can contribute to various diseases, including obesity [1].

This review article explores the complex relationship between gut microbiota and obesity. It provides a detailed overview of obesity, introduces the importance of gut microbiota, and explains the mechanisms involved, such as energy metabolism, appetite regulation, inflammation, and the gut-brain axis. The article also delves into influential factors like diet, lifestyle, antibiotics, and early-life influences, highlighting their impact on gut microbiota in the context of obesity. Additionally, it discusses various therapeutic interventions, like dietary modifications, prebiotics, probiotics, fecal microbiota transplantation (FMT), and personalized treatment approaches. The review also covers clinical applications, future research directions, and ethical considerations, making it a comprehensive guide to the topic.

The relationship between the gut microbiota and obesity is complex. An altered gut microbiota can affect the absorption, storage, and expenditure of energy from the diet, disrupting the body's energy balance. It also impacts the control of food intake through hormones that influence metabolic functions and brain regions responsible for eating behavior. This two-way communication, known as the microbiota-gut-brain axis, plays a crucial role in balancing appetite, storage, and energy expenditure [3]. Interestingly, disruptions in gut microbiota composition and diversity are shared between individuals with obesity and those experiencing undernutrition [4,5]. This highlights the importance of gut microbiota in maintaining a healthy body weight and fat storage. However, ongoing research is needed to determine whether gut dysbiosis is a root cause or a consequence of obesity and undernutrition [3]. It is important to note that humans have always had a natural inclination to protect their energy stores throughout evolution. This may provide insight into the obesity epidemic in developed nations, as energy-dense foods have become more readily available over time. The gut microbiota has also evolved to assist with various functions in the body, including nutrient synthesis and the efficient acquisition, storage, and utilization of energy from the diet [6].

## Review

Gut microbiota and obesity

Obesity is a global epidemic characterized by the accumulation of excessive body fat. It's identified using the body mass index (BMI), which measures weight in relation to height. In adults, a BMI ranging from 25.0 to 29.9 kg/m^2^ is considered overweight, while a BMI of 30 kg/m^2^ or higher is categorized as obese [7]. Presently, there are around 650 million adults and approximately 340 million children and adolescents aged five to 19 who are grappling with obesity [8]. The global incidence of obesity has doubled since 1980, reaching a point where almost one-third of the global population is now categorized as overweight or obese [9]. Obesity affects both genders, but it is more prevalent among women and older individuals. This complex condition is influenced by various factors, including genetics, environment, and social determinants [8].

In recent years, there has been a growing interest in understanding the role of gut microbiota in obesity. The gut microbiota mainly consists of bacteria but also includes fungi, parasites, archaea, and viruses. This dynamic community has a vast genetic and functional profile that significantly exceeds the human genome [10]. It plays a crucial role in maintaining both gut and overall human health [11]. The link between gut microbiota and obesity is becoming increasingly evident. Studies suggest that dietary patterns can shape the composition of gut flora. Diets high in fat and carbohydrates tend to promote the growth of harmful bacteria while decreasing the number or diversity of beneficial microbes. These shifts in microbial composition can lead to various consequences, such as compromised gut barrier function, inflammation, dyslipidemia, and hormonal imbalances, ultimately contributing to the development of obesity, diabetes, and even cancer [12]. Individuals with obesity often exhibit imbalances in their gut microbiota, indicating a potential connection between the makeup of the gut flora and the development of obesity [12]. This link has piqued considerable interest within the scientific community, as gut microbiota has been associated with obesity as well as luminal conditions like inflammatory bowel diseases and irritable bowel syndrome [11].

Mechanisms of gut microbiota in obesity

Obesity has garnered significant attention for its intricate connection with the gut microbiota. The human gut microbiome is predominantly composed of the *Firmicutes* and *Bacteroidetes *phyla, together constituting 90% of the total community, with smaller contributions from *Proteobacteria*, *Actinobacteria*, and *Verrucomicrobia*. Among the *Firmicutes*, notable genera include *Lactobacillus*, *Bacillus*, *Clostridium*, *Enterococcus*, and *Ruminococcus*, while the *Bacteroides *and *Prevotella *genera dominate the *Bacteroidetes *phylum. Studies involving the transplantation of gut microbiota have demonstrated significant effects on body fat in both mice and humans, establishing a connection between obesity and variations in microbiome diversity, phyla composition, and ratios [13].

The gut microbiota exerts a significant influence on our energy metabolism, affecting various aspects of our well-being. It plays a vital role in the absorption of nutrients and the extraction of energy from our diet, ultimately influencing our ability to utilize dietary energy. This, in turn, contributes to the regulation of fat storage and the synthesis of essential gut peptides like GLP-1 and PYY, which are crucial for maintaining energy balance [14]. Moreover, the gut microbiota has a pivotal role in regulating appetite and satiety, influencing our food intake through diverse mechanisms, including the release of satiety peptides and the modulation of reward pathways [15]. Furthermore, the gut microbiota's impact on inflammation and insulin resistance is substantial. High-fat diets can lead to conditions such as obesity, inflammation, insulin resistance, and type 2 diabetes, in part due to the interactions between dietary components, the gut microbiota, and the innate immune system [14]. The presence of bacterial lipopolysaccharide (LPS) from intestinal flora can trigger chronic subclinical inflammation and insulin resistance, further establishing a connection between the gut microbiota and metabolic disorders [16].

A sophisticated feedback system exists between the gut and the brain, often referred to as the gut-brain axis. The gut-brain axis plays a pivotal role in energy homeostasis. Microbiota-driven changes in gut-brain signaling affect both the homeostatic and hedonic aspects of feeding regulation. Satiety, inflammation, and modulation of reward pathways are all influenced by the gut microbiota. This intricate relationship between the gut and the brain contributes significantly to the development of obesity and metabolic diseases [17]. Genetic factors are integral to the obesity-gut microbiota interplay. Genetic variations impact the composition and diversity of the gut microbiota, with specific genes influencing the presence and abundance of certain bacteria. For example, variations in genes related to immune regulation and obesity correlate with gut microbiota composition. Furthermore, maternal obesity during pregnancy has lasting effects on the child's gut microbiota. The transmission of the gut microbiota from mother to child can result in metabolic disorders in the offspring. Maternal obesity is linked to dysbiosis in the gut microbiota of the child, reinforcing the risk of obesity in children with obese mothers [18].

Influential factors shaping gut microbiota in obesity

The gut microbiota plays a pivotal role in the maintenance of overall health, particularly its involvement in obesity. Several influential factors shape the gut microbiota composition, leading to a deeper understanding of its role in this widespread health concern. Diet significantly shapes the gut microbiota's role in obesity. Circadian rhythms, aligned with daily eating and fasting patterns, interact with the microbiota, influencing and responding to its cues. The immune system works with the microbiota to synchronize these circadian clocks. The gut microbiome affects daily rhythms in the digestive system, integrating signals from nutrients, hormones, and the immune system [19,20]. Carbohydrates that are easily digested become glucose in the small intestine, while indigestible dietary fiber, such as microbiota-accessible carbohydrates (MACs), reach the large intestine. Colon bacteria efficiently ferment these fibers into short-chain fatty acids (SCFAs), like acetate, propionate, and butyrate. SCFAs are key in preventing diet-induced obesity and promoting colon health [20,21]. Feeding the gut microbiota with dietary fiber also safeguards against diet-induced obesity by revitalizing colon health through IL-22 [22]. Dietary fat, both in quantity and quality, impacts gut microbiota composition. High fat intake, particularly saturated fats, reduces microbial diversity and richness, linked to cardiometabolic risks [20,23,24]. Proteins also influence microbiota composition, where factors like amino acid makeup, source, and digestibility are critical. Animal-protein-rich diets relate to specific bacteria and microbial metabolite changes, while plant-based proteins foster beneficial bacteria like *Lactobacilli *and *Bifidobacteria*, enhancing SCFA production [20]. These dietary factors collectively shape the complex interplay between diet, microbiota, and obesity.

Lifestyle choices, such as sedentary behavior and a lack of physical activity, can further influence gut microbiota composition. Smoking and lack of exercise have been linked to an increased risk of colorectal cancer and can impact the large bowel, potentially disrupting microbial populations [25,26]. Conversely, exercise has been shown to increase microbial diversity, particularly in professional athletes who follow a specialized diet, underlining the connection between physical activity and gut health [27]. Antibiotics and medications can significantly affect the gut microbiota. Antibiotics, in particular, have been associated with a reduction in bacterial diversity and may lead to a dysbiotic gut microbiome. Such alterations in microbial populations are thought to contribute to obesity through mechanisms like increased energy extraction from indigestible polysaccharides, changes in hepatic lipogenesis, metabolic signaling, and reduced intestinal defense and immunity [28]. Early-life factors also play a crucial role in the developmental origins of obesity. Maternal smoking during pregnancy, high gestational weight gain, and Caesarian section have been identified as risk factors for childhood obesity. Parental weight status, particularly maternal and paternal BMI, has emerged as the most significant risk factor. Breastfeeding for an extended period (four to 11 months) has been found to be protective against childhood obesity, highlighting the importance of early-life nutrition and parental influences [29].

Therapeutic interventions targeting gut microbiota

Emerging evidence highlights the profound influence that the gut microbiota exerts on human health, ranging from metabolic processes to immune function. Understanding and modulating the gut microbiota has, therefore, become a focal point in the quest for innovative therapeutic interventions. It is reasonable to hypothesize that modulating the gut microbiota through external approaches, such as diet, may offer substantial benefits to the host. Among these approaches, dietary strategies are desirable due to their cost-effectiveness and safety. Both probiotics and prebiotics are promising in this regard, given their direct influence on the gut microbiota [30].

Prebiotics are dietary fibers selectively fermented by gut bacteria, prompting specific changes in the gut microbiota's composition and activity that benefit host health [31]. Inulin-type prebiotics have been demonstrated to have various positive effects on human physiology. They induce satiety, influence gut peptides involved in appetite regulation, and promote the growth of beneficial bacteria like *Bifidobacterium *and *Lactobacillus *[32-35]. In addition to these effects, prebiotics have been shown to selectively modulate the gut microbiota, leading to changes in the populations of various bacterial species [30]. Another example is flaxseed, which contains a substantial amount of dietary fibers, including soluble viscous fibers known as mucilage. Consumption of these fibers has been linked to beneficial effects on glucose homeostasis and lipid metabolism in human subjects, demonstrating the potential of dietary interventions in modulating the gut microbiota's function and structure [36]. Probiotics, commonly consisting of microorganisms like *Lactobacillus *species, have garnered attention for their potential to impart health benefits when ingested. These living microorganisms can exert their influence directly through interactions with host cells or indirectly by affecting other bacterial species within the gut microbiota. For instance, *Lactobacillus paracasei *has been associated with a healthy metabolic profile [36-38]. In one study, the administration of *Lactobacillus salivarius* Ls-33 to obese adolescents resulted in an increased ratio of Bacteroides-Prevotella-Porphyromonas group to Firmicutes-related bacteria. This shift in bacterial composition was associated with a positive impact on body weight [39]. Furthermore, a combined intervention involving probiotics and herbal medicine led to reductions in body weight and waist circumference, underscoring the role of probiotics in deterring obesity by reducing LPS production through alterations in the gut microbiota [40]. These findings highlight the potential of probiotics and prebiotics in reshaping the gut microbiota to promote weight loss and metabolic improvements.

In addition to probiotics and prebiotics, low-calorie and high-fiber diets have gained recognition as effective therapeutic interventions targeting gut microbiota. Very-low-calorie ketogenic Diets (VLCKD), characterized by carbohydrate deprivation and the induction of ketosis, have shown potential in modulating the gut microbiota and promoting weight loss [41]. One study involving women on a VLCKD reported significant reductions in body weight and improvements in body composition parameters after 45 days of dietary intervention [42]. Furthermore, another trial investigated the effects of a VLCKD combined with a symbiotic on the microbiota of adults with obesity, revealing significant changes in the gut microbiota diversity and the abundance of specific bacterial genera [43]. This evidence suggests that VLCKD could potentially reverse obesity-associated dysbiosis, highlighting the dynamic interplay between diet and gut microbiota.

High-fiber diets, including those common in rural, unindustrialized, Mediterranean, or vegetarian diets, have consistently demonstrated the ability to shape the gut microbiota. Individuals adhering to fiber-rich diets exhibit distinctive microbial community structures compared to their counterparts living in developed areas [44]. Recent studies have emphasized the significant correlation between dietary habits, such as the intake of whole grains and vegetables, and changes in the gut microbiome [45]. While some research suggests that agrarian diets high in fruit and legume fiber enhance microbial richness, the Western diet's decreased fiber intake is thought to diminish intestinal biodiversity [44]. These findings underscore the complex interplay between diet and gut microbiota diversity.

Among the innovative approaches in this field, FMT stands out as an emerging therapy with great potential. FMT is a microbial-based strategy aimed at restoring disrupted gut microbial ecosystems, a concept that has gained traction due to its efficacy in treating conditions like *Clostridium difficile* infection (CDI) [46]. Clinical trials have shed light on the potential benefits of FMT for adults with obesity and metabolic syndromes, suggesting it could open new pathways for personalized metabolic maintenance and weight loss preservation [47]. The link between gut microbiota and obesity has led to investigations into the transplantation of gut microbiota from lean and healthy donors into obese and metabolic syndrome recipients [46]. These studies highlight the potential for FMT to influence metabolic outcomes, but more research is needed to establish its clinical utility fully. An alternative to FMT is Bacterial Consortium Therapy (BCT), which offers a more targeted and controlled approach to gut microbiota modulation. BCT utilizes well-defined drug compositions derived from clonally isolated bacteria to trigger specific immune responses and modulate the intestinal ecosystem. Research has shown that BCT can provide results comparable to FMT, with the advantage of precise control over the bacterial consortium used, ensuring safety by minimizing the risk of pathogenic microbes. BCT can effectively address different levels and types of dysbiosis, making it a safer alternative to FMT for modulating intestinal dysbiosis [48].

Personalized obesity treatments, informed by a comprehensive -omic profiling encompassing metabolomics, genomics, transcriptomics, and gut microbiome analysis, provide a more precise grasp of the condition. Rather than categorizing individuals solely as “obese,” this multifaceted approach can unveil distinctive biomarkers that offer a deeper insight into their metabolic state. Identifying an individualized -omic signature paves the way for personalized prognostic, diagnostic, and therapeutic strategies, aiding in symptom monitoring, treatment effectiveness, potential drug-related side effects, and susceptibility to relapse [49]. Additionally, precision nutrition tailors dietary guidance to an individual's unique internal and external factors, potentially playing a pivotal role in disease prevention and maintaining general well-being by recommending specific diets based on individual parameters. To optimize precision nutrition, gathering data on the individual's gut microbiome and their specific food responses is essential. This enhances the customization of dietary recommendations for future well-being [50].

While microbiome therapeutics hold promise, they face a multitude of challenges. The primary hurdle is the selection of suitable microbes to tackle complex diseases. It is essential to thoroughly characterize these microbes based on their functional advantages when considering them for treatment. The effectiveness of microbiome therapeutics, often demonstrated in rodent models, requires rigorous human trials for clinical adaptation. The stability and resilience of clinically relevant microbial strains are critical for their successful application in therapy. Understanding the environmental conditions and intricate interactions among diverse microbial species is vital for optimizing their functionality. Furthermore, safety and regulatory issues are of paramount concern [51]. The potential horizontal transfer of recombinant DNA from the engineered microbiome to the native microbiome raises significant concerns. Likewise, the release of recombinant probiotics into the environment could have adverse consequences [52,53].

A well-structured regulatory framework is necessary to ensure the safety of microbiome therapeutics, preventing potential adverse effects and unintended environmental release of engineered microbes [51]. A comprehensive grasp of the factors influencing host-associated microbial communities and governing host-bacterial interactions is imperative for the informed design of microbiome therapeutics. Achieving the vision of fully autonomous cellular therapies depends on the creation of clinically applicable biosensors and resilient, dependable genetic circuits. Furthermore, being mindful of biocontainment, and safety concerns in novel therapeutic research is essential for expediting the translation of fundamental research into clinical practice [54].

Clinical applications and future directions

Recent breakthroughs in gut microbiota research are poised to revolutionize clinical practice and shape the future of healthcare. Cutting-edge technologies such as next-generation sequencing, untargeted metabolomics, and advanced bioinformatics have empowered researchers to discern the precise functions of various bacterial taxa within the microbiome. This newfound understanding allows for the identification of specific target species for microbiota-based therapeutics, a development that holds immense promise. One notable area of exploration is the translation of this research into clinical applications, with a focus on personalized and condition-based microbiome-related treatments. As bioinformatics pipelines and machine learning algorithms continually evolve, the integration of omics data into clinical practice becomes more refined. The emergence of microbiota-based diagnostics and customized probiotics tailored to an individual's unique microbiota profile presents a potential paradigm shift in healthcare [55].

The prospects for microbiota-based therapies in addressing obesity are highly encouraging. Exploring the influence of diet, including prebiotics, on shaping a more advantageous microbial community within the gut is a significant research avenue [56]. Prospective studies featuring extended follow-up periods, emphasizing the shifts in microbial composition and its correlation with the health of the host, have the potential to validate the intestinal microbiota as a valuable biomarker for health and various disease states. To advance in this field, crucial technological innovations, particularly in nucleic acid sequencing, computational infrastructure, and bioinformatics, are necessary. These innovations empower researchers to delve into both the structural and functional aspects of the microbiome, offering the essential tools for data analysis and interpretation [57]. As the global population continues to age, there's a growing quest for novel, cost-effective, and low-risk therapeutic options. Interventions like fecal microbiota transplants are likely to proliferate as autonomous treatments, complementary therapies, and preventive measures in the ongoing battle against obesity and associated metabolic disorders [55].

## Conclusions

This narrative review provides a comprehensive exploration of the intricate relationship between gut microbiota and obesity. Obesity, a global health concern with far-reaching implications, is influenced by the composition and activity of the gut microbiota through multiple mechanisms, including energy metabolism, appetite regulation, inflammation, and the gut-brain axis. The review has highlighted the significance of dietary choices, lifestyle decisions, antibiotics and medications, and early-life experiences as influential factors shaping the gut microbiota and its connection to obesity. The gut microbiota is a dynamic entity that can be influenced by external factors, providing a promising avenue for interventions aimed at mitigating obesity and related metabolic disorders. The therapeutic interventions targeting the gut microbiota, such as dietary modifications through prebiotics and probiotics, fecal microbiota transplantation, bacterial consortium therapy, and precision nutrition, have the potential to reshape the gut microbiota, positively influencing weight regulation and metabolic health. These interventions offer a path toward personalized treatment approaches and innovative strategies for managing obesity. While there are challenges and limitations, such as identifying microbial signatures and addressing safety and regulatory concerns, the development of microbiota-based therapeutics represents a promising frontier in healthcare. The article also underscores the clinical applications and future directions of microbiota research, emphasizing the translation of scientific findings into practical healthcare solutions. The potential to use microbiota-based diagnostics, customized probiotics, and personalized interventions holds promise for improving public health and individual well-being. However, ethical considerations and safety concerns remain paramount as this field continues to evolve.

## References

[1] Cheng Z, Zhang L, Yang L, Chu H (2022). The critical role of gut microbiota in obesity. Front Endocrinol (Lausanne).

[2] Nicholson JK, Holmes E, Kinross J, Burcelin R, Gibson G, Jia W, Pettersson S (2012). Host-gut microbiota metabolic interactions. Science.

[3] de Clercq NC, Groen AK, Romijn JA, Nieuwdorp M (2016). Gut microbiota in obesity and undernutrition. Adv Nutr.

[4] Ley RE, Turnbaugh PJ, Klein S, Gordon JI (2006). Microbial ecology: human gut microbes associated with obesity. Nature.

[5] Gordon JI, Dewey KG, Mills DA, Medzhitov RM (2012). The human gut microbiota and undernutrition. Sci Transl Med.

[6] Krajmalnik-Brown R, Ilhan ZE, Kang DW, DiBaise JK (2012). Effects of gut microbes on nutrient absorption and energy regulation. Nutr Clin Pract.

[7] Apovian CM (2016). Obesity: definition, comorbidities, causes, and burden. Am J Manag Care.

[8] Sørensen TI, Martinez AR, Jørgensen TS (2022). Epidemiology of obesity. Handb Exp Pharmacol.

[9] Chooi YC, Ding C, Magkos F (2019). The epidemiology of obesity. Metabolism.

[10] Al Bander Z, Nitert MD, Mousa A, Naderpoor N (2020). The gut microbiota and inflammation: an overview. Int J Environ Res Public Health.

[11] Jandhyala SM, Talukdar R, Subramanyam C, Vuyyuru H, Sasikala M, Nageshwar Reddy D (2015). Role of the normal gut microbiota. World J Gastroenterol.

[12] Noor J, Chaudhry A, Batool S, Noor R, Fatima G (2023). Exploring the impact of the gut microbiome on obesity and weight loss: a review article. Cureus.

[13] Pinart M, Dötsch A, Schlicht K (2021). Gut microbiome composition in obese and non-obese persons: a systematic review and meta-analysis. Nutrients.

[14] Cani PD, Delzenne NM (2009). The role of the gut microbiota in energy metabolism and metabolic disease. Curr Pharm Des.

[15] Rautmann AW, de La Serre CB (2021). Microbiota’s role in diet-driven alterations in food intake: satiety, energy balance, and reward. Nutrients.

[16] Saad MJ, Santos A, Prada PO (2016). Linking gut microbiota and inflammation to obesity and insulin resistance. Physiology (Bethesda).

[17] van Son J, Koekkoek LL, La Fleur SE, Serlie MJ, Nieuwdorp M (2021). The role of the gut microbiota in the gut-brain axis in obesity: mechanisms and future implications. Int J Mol Sci.

[18] Liu BN, Liu XT, Liang ZH, Wang JH (2021). Gut microbiota in obesity. World J Gastroenterol.

[19] Zheng D, Ratiner K, Elinav E (2020). Circadian influences of diet on the microbiome and immunity. Trends Immunol.

[20] Nova E, Gómez-Martinez S, González-Soltero R (2022). The influence of dietary factors on the gut microbiota. Microorganisms.

[21] Galanakis CM (2019). Dietary fiber: properties, recovery, and applications. Academic Press.

[22] Zou J, Chassaing B, Singh V (2018). Fiber-mediated nourishment of gut microbiota protects against diet-induced obesity by restoring IL-22-mediated colonic health. Cell Host Microbe.

[23] Wolters M, Ahrens J, Romaní-Pérez M (2019). Dietary fat, the gut microbiota, and metabolic health - a systematic review conducted within the MyNewGut project. Clin Nutr.

[24] Wan Y, Wang F, Yuan J (2019). Effects of dietary fat on gut microbiota and faecal metabolites, and their relationship with cardiometabolic risk factors: a 6-month randomised controlled-feeding trial. Gut.

[25] Huxley RR, Ansary-Moghaddam A, Clifton P, Czernichow S, Parr CL, Woodward M (2009). The impact of dietary and lifestyle risk factors on risk of colorectal cancer: a quantitative overview of the epidemiological evidence. Int J Cancer.

[26] Conlon MA, Bird AR (2014). The impact of diet and lifestyle on gut microbiota and human health. Nutrients.

[27] Clarke SF, Murphy EF, O'Sullivan O (2014). Exercise and associated dietary extremes impact on gut microbial diversity. Gut.

[28] Leong KS, Derraik JG, Hofman PL, Cutfield WS (2018). Antibiotics, gut microbiome and obesity. Clin Endocrinol (Oxf).

[29] Bammann K, Peplies J, De Henauw S (2014). Early life course risk factors for childhood obesity: the IDEFICS case-control study. PLoS One.

[30] Dahiya DK, Renuka Renuka, Puniya M (2017). Gut microbiota modulation and its relationship with obesity using prebiotic fibers and probiotics: a review. Front Microbiol.

[31] Gibson GR, Scott KP, Rastall RA (2010). Dietary prebiotics: current status and new definition. Food Sci Technol Bull Funct Foods.

[32] Cani PD, Joly E, Horsmans Y, Delzenne NM (2006). Oligofructose promotes satiety in healthy human: a pilot study. Eur J Clin Nutr.

[33] Cani PD, Lecourt E, Dewulf EM (2009). Gut microbiota fermentation of prebiotics increases satietogenic and incretin gut peptide production with consequences for appetite sensation and glucose response after a meal. Am J Clin Nutr.

[34] Parnell JA, Reimer RA (2009). Weight loss during oligofructose supplementation is associated with decreased ghrelin and increased peptide YY in overweight and obese adults. Am J Clin Nutr.

[35] Gibson GR, Probert HM, Loo JV, Rastall RA, Roberfroid MB (2004). Dietary modulation of the human colonic microbiota: updating the concept of prebiotics. Nutr Res Rev.

[36] Brahe LK, Le Chatelier E, Prifti E (2015). Dietary modulation of the gut microbiota--a randomised controlled trial in obese postmenopausal women. Br J Nutr.

[37] Gordon JI (2012). Honor thy gut symbionts redux. Science.

[38] de Vrese M, Schrezenmeir J (2008). Probiotics, prebiotics, and synbiotics. Adv Biochem Eng Biotechnol.

[39] Larsen N, Vogensen FK, Gøbel RJ, Michaelsen KF, Forssten SD, Lahtinen SJ, Jakobsen M (2013). Effect of Lactobacillus salivarius Ls-33 on fecal microbiota in obese adolescents. Clin Nutr.

[40] Lee SJ, Bose S, Seo JG, Chung WS, Lim CY, Kim H (2014). The effects of co-administration of probiotics with herbal medicine on obesity, metabolic endotoxemia and dysbiosis: a randomized double-blind controlled clinical trial. Clin Nutr.

[41] Zambrano AK, Cadena-Ullauri S, Guevara-Ramírez P (2023). The impact of a very-low-calorie ketogenic diet in the gut microbiota composition in obesity. Nutrients.

[42] Barrea L, Verde L, Santangeli P (2023). Very low-calorie ketogenic diet (VLCKD): an antihypertensive nutritional approach. J Transl Med.

[43] Gutiérrez-Repiso C, Hernández-García C, García-Almeida JM (2019). Effect of synbiotic supplementation in a very-low-calorie ketogenic diet on weight loss achievement and gut microbiota: a randomized controlled pilot study. Mol Nutr Food Res.

[44] Fu J, Zheng Y, Gao Y, Xu W (2022). Dietary fiber intake and gut microbiota in human health. Microorganisms.

[45] Zhang Y, Chen H, Lu M, Cai J, Lu B, Luo C, Dai M (2022). Habitual diet pattern associations with gut microbiome diversity and composition: results from a Chinese adult cohort. Nutrients.

[46] Zhang Z, Mocanu V, Cai C (2019). Impact of fecal microbiota transplantation on obesity and metabolic syndrome-a systematic review. Nutrients.

[47] Biazzo M, Deidda G (2022). Fecal microbiota transplantation as new therapeutic avenue for human diseases. J Clin Med.

[48] Gill VJ, Soni S, Shringarpure M (2022). Gut microbiota interventions for the management of obesity: a literature review. Cureus.

[49] Gawlik A, Salonen A, Jian C (2021). Personalized approach to childhood obesity: lessons from gut microbiota and omics studies. Narrative review and insights from the 29th European childhood obesity Congress. Pediatr Obes.

[50] Song EJ, Shin JH (2022). Personalized diets based on the gut microbiome as a target for health maintenance: from current evidence to future possibilities. J Microbiol Biotechnol.

[51] Yadav M, Chauhan NS (2022). Microbiome therapeutics: exploring the present scenario and challenges. Gastroenterol Rep (Oxf).

[52] Smillie CS, Smith MB, Friedman J, Cordero OX, David LA, Alm EJ (2011). Ecology drives a global network of gene exchange connecting the human microbiome. Nature.

[53] Ceroni F, Algar R, Stan GB, Ellis T (2015). Quantifying cellular capacity identifies gene expression designs with reduced burden. Nat Methods.

[54] Mimee M, Citorik RJ, Lu TK (2016). Microbiome therapeutics - advances and challenges. Adv Drug Deliv Rev.

[55] Turjeman S, Koren O (2022). Using the microbiome in clinical practice. Microb Biotechnol.

[56] Brownawell AM, Caers W, Gibson GR, Kendall CW, Lewis KD, Ringel Y, Slavin JL (2012). Prebiotics and the health benefits of fiber: current regulatory status, future research, and goals. J Nutr.

[57] Bäckhed F, Fraser CM, Ringel Y (2012). Defining a healthy human gut microbiome: current concepts, future directions, and clinical applications. Cell Host Microbe.

